# Incidence and impact of odontogenic infection-related febrile episodes during perioperative doxorubicin-cyclophosphamide or docetaxel-cyclophosphamide chemotherapy for breast cancer

**DOI:** 10.1007/s00520-026-10977-4

**Published:** 2026-07-09

**Authors:** Yoshihiko Soga, Kumiko Matsuzaki, Hirotaka Kosaki, Tomoko Kishimoto, Aiko Yoshitomi, Makoto Kajizono, Takashi Makita, Yuta Tanaka, Yoshito Zamami, Tadahiko Shien, Hiroyoshi Doihara

**Affiliations:** 1 Division of Hospital Dentistry, Okayama University Hospital, 2-5-1, Shikata-Cho, Kita-Ku, Okayama, 700-8558 Japan; 2 Department of Pharmacy, Okayama University Hospital, Okayama, Japan; 3 Department of Pharmacy, Okayama City Hospital, Okayama, Japan; 4 Department of Breast and Endocrine Surgery, Okayama University Hospital, Okayama, Japan; 5 Department of Surgery, Kawasaki Medical School General Medical Center, Okayama, Japan

**Keywords:** Breast cancer, Perioperative chemotherapy, Odontogenic infection, Oral infection, Febrile episodes, Relative dose intensity

## Abstract

**Background:**

Doxorubicin-cyclophosphamide (AC) and docetaxel-cyclophosphamide (TC) are standard perioperative chemotherapy regimens for breast cancer. Febrile neutropenia (FN) is a common adverse event, and odontogenic infections may contribute to febrile episodes. This study was performed to investigate the incidence and impact of febrile episodes clinically associated with odontogenic infections during AC and TC chemotherapy.

**Methods:**

A total of 408 patients with breast cancer receiving 4 cycles of AC (*n* = 285) or TC (*n* = 123) between 2015 and 2020 were included in this single-center retrospective study. Patients recorded daily axillary temperatures and oral symptoms. Dental evaluations were performed when oral infection was suspected. Febrile episodes (≥ 37.5 °C) were assessed, and their clinical association with odontogenic infections was determined based on dental records and the documented clinical course. Relative dose intensity (RDI) was calculated to assess chemotherapy delivery.

**Results:**

Overall, 40.2% of patients experienced febrile episodes, and the frequency was higher in the TC group (48.8%) than the AC group (36.5%, *P* = 0.02). Febrile episodes associated with oral infections occurred in 9.1% of patients, of which 78.4% were clinically associated with odontogenic infections (7.1% of all patients). Most such episodes occurred during the first chemotherapy cycle (79.3%, *P* < 0.01). Chemotherapy RDI was reduced in three patients with febrile episodes clinically associated with odontogenic infection, but remained ≥ 85%.

**Conclusions:**

Febrile episodes clinically associated with odontogenic infections occurred in approximately 7%–8% of patients with breast cancer receiving perioperative AC or TC chemotherapy, predominantly during the first cycle. Odontogenic infections should be considered one possible source of febrile episodes during treatment.

## Introduction

Doxorubicin-cyclophosphamide (AC) and docetaxel-cyclophosphamide (TC) are major perioperative chemotherapy regimens for breast cancer. Before the introduction of taxanes in the 1990 s, no 4-cycle chemotherapy regimen had proven superior to AC. AC delivered in 4 cycles remains a standard form of adjuvant therapy, often followed by taxanes. A comparative trial of preoperative versus postoperative adjuvant chemotherapy with AC found no differences in disease-free survival (DFS) or overall survival (OS), and both regimens showed equivalent efficacy in improving prognosis [1]. Therefore, AC is used in both pre- and postoperative chemotherapy. TC delivered in 4 cycles is also considered standard adjuvant therapy, with efficacy demonstrated by a randomized controlled trial [2, 3]. A comparison of AC and TC as postoperative adjuvant chemotherapies for Stage I, II, and III resectable invasive breast cancer, showed significantly superior DFS and OS for TC therapy [2, 3].

Febrile neutropenia (FN) is a common adverse event associated with both AC and TC chemotherapy. The National Comprehensive Cancer Network (NCCN) clinical guidelines classify AC regimens (60/600 mg/m^2^, 4 cycles at 3-week intervals) as intermediate risk for FN (10%−20%), while classifying TC regimens (75/600 mg/m^2^, 4 cycles at 3-week intervals) as high risk for FN (> 20%) [4]. Most chemotherapy regimens, including AC, cause neutropenic nadirs between days 10 and 14, whereas the TC regimen is characterized by an early neutrophil nadir, typically around day 8, with a substantial decrease in neutrophil count and elevated risk of FN [5]. Appropriate use of granulocyte colony-stimulating factor (G-CSF) support and antibiotics is important [4, 6, 7].

In daily clinical practice, FN has been suggested to be associated with odontogenic infections. The impact of odontogenic infection on FN is recognized in theory. However, most studies have focused on FN in the setting of ultimate bone marrow suppression, such as hematopoietic stem cell transplantation. A recent review concluded that there is limited information on patients treated with myelosuppressive chemotherapy for solid tumors [8].

At our institute, both AC and TC regimens are typically administered on an outpatient basis following initial inpatient administration. At each outpatient chemotherapy visit, physicians, nurses, or pharmacists inquire about adverse events, including fever and oral symptoms, and these findings are documented in the medical records. When symptoms suggestive of oral infection occur, dentists provide dental examination and management during the phase of neutrophil recovery. As patients are usually at home, it is not possible to determine precisely whether the neutrophil count at the time of fever met the criteria for FN. However, patients record their body temperature daily, allowing confirmation of whether febrile episodes have occurred.

This study was performed to clarify the incidence and impact of odontogenic infections on febrile episodes during AC and TC perioperative chemotherapy for breast cancer.

## Subjects and methods

### Study design

This single-center retrospective study was conducted at the Division of Hospital Dentistry, the Department of Breast and Endocrine Surgery, and the Department of Pharmacy at Okayama University Hospital.

### Subjects

A total of 408 consecutive patients who completed either 4 cycles of AC or TC therapy described below as perioperative chemotherapy for breast cancer at our institution between April 1, 2015, and March 31, 2020, were enrolled in the study.

### Chemotherapy regimens

The chemotherapy regimens administered to patients in this study were as follows: AC (doxorubicin 60 mg/m^2^ IV on day 1; cyclophosphamide 600 mg/m^2^ IV on day 1; every 21 days for 4 cycles) and TC (docetaxel 75 mg/m^2^ IV on day 1; cyclophosphamide 600 mg/m^2^ IV on day 1; every 21 days for 4 cycles). AC was generally administered as part of the AC followed by paclitaxel regimen (AC-T). Patients with a low risk of recurrence or with estrogen receptor (ER)-positive tumors expected to respond to endocrine therapy, who do not require full AC-T regimens but for whom adjuvant chemotherapy is still indicated, were treated with TC regimens [9].

The initial treatment was given during hospitalization. After confirming the lack of adverse events, especially allergic reactions, the patient was discharged the next day. The second through fourth treatments were administered as outpatient chemotherapy.

### Infection control

Oral antibiotic (ciprofloxacin) was prescribed at the start of chemotherapy as a countermeasure against FN. Patients were instructed to take the oral antibiotic for 5 days if they recorded an axillary temperature of 37.5 °C or higher after beginning chemotherapy.

Preventive G-CSF was generally not administered. Its use was determined by the attending physician according to the following criteria:

i. Development of fever (≥ 38.0 °C) with absolute neutrophil count (ANC) < 1000/mm^3^ or 500/mm^3^ after completion of chemotherapy, and.
ii. For subsequent cycles of the same chemotherapy after meeting condition (i), G-CSF administration was initiated when ANC reached 1000/mm^3^.

### Oral management

At the initiation of chemotherapy, patients receive education on the importance of oral hygiene from physicians, nurses, and pharmacists. However, due to limited manpower, routine dental examinations were not performed for all patients before chemotherapy. At each outpatient chemotherapy visit, physicians, nurses, or pharmacists inquired about adverse events, including fever and oral symptoms, and these findings were documented in the medical records. When symptoms suggestive of an oral infection occurred, almost all patients were referred to the Division of Hospital Dentistry at the same institute or to their regular dentist for dental examination and management.

### Febrile episode assessment

Patients recorded daily axillary temperature and other adverse events in a notebook. Medical staff documented the patient’s fever status during the period of chemotherapy in the medical records based on these records and interviews with the patient. Febrile episodes were defined as axillary temperature ≥ 37.5 °C according to the FN guidelines of Japan [10].

### Association between febrile episodes and oral infection

The clinical association between febrile episodes and oral infection was assessed from dental records and the documented clinical course. For patients managed at external dental clinics or who did not attend a dentist, study dentists reviewed the available medical records to assess the likelihood of oral infection at the time of a febrile episode. Microbiological confirmation was not systematically available.

### Relative dose intensity

Relative dose intensity (RDI) was calculated as the ratio of delivered dose intensity to planned dose intensity, expressed as a percentage. Dose intensity was defined as the cumulative dose per unit time divided by the planned cumulative dose per unit time for each regimen.

### Statistical analysis

Statistical analyses were performed using Stata 19 (StataCorp, College Station, TX, USA). In all analyses, *P* < 0.05 was taken to indicate statistical significance.

## Results

### Patient characteristics

All patients (*n* = 408) were female with a median age of 51 years. Of these, 285 patients received AC, with a median age of 51 years (range 23–78 years) and 123 received TC, with a median age of 50 years (range 31–74 year). There was no significant difference in age between the AC and TC groups (*P* = 0.68, Wilcoxon’s rank-sum test).

### Frequency of febrile patients

**Fig. 1:**
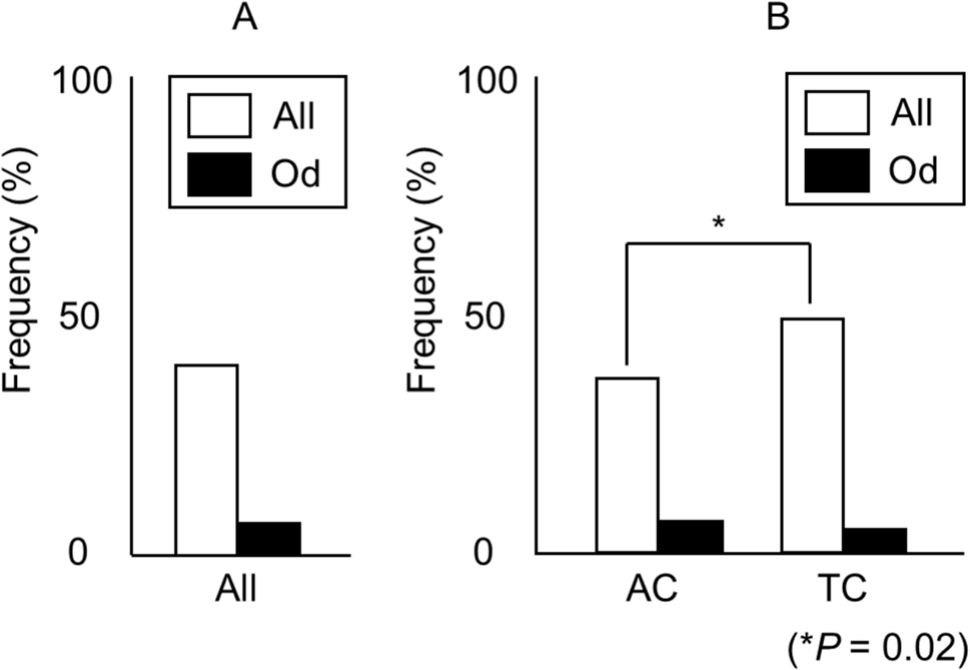
Febrile patients during the chemotherapy period. **A** All subjects. Febrile episodes occurred in 40.2% (164/408) of patients, and 7.1% (29/408) had febrile episodes clinically associated with odontogenic infections. **B** By chemotherapy type (AC vs. TC). The overall febrile rate was higher in the TC group (48.8%, 60/123) than the AC group (36.5%, 104/285) (*P* = 0.02, χ^2^ test). There was no significant difference in occurrence of febrile episodes clinically associated with odontogenic infections between the AC group (7.7%, 22/285) and the TC group (5.7%, 7/123) (*P* = 0.46, χ^2^ test)

The frequencies of patients who developed febrile episodes are shown in Fig. 1.

#### All febrile patients

Overall, 40.2% (164/408) of patients developed at least one febrile episode (Fig. 1A). The frequency was significantly higher in the TC group (48.8%, 60/123) than the AC group (36.5%, 104/285) (*P* = 0.02, χ^2^ test) (Fig. 1B).

#### Febrile patients related to oral cavity infections

Among all patients, 9.1% (37/408) developed febrile episodes clinically associated with oral cavity infections. There was no significant difference between the AC group (9.1%, 26/285) and the TC group (8.9%, 11/123) (*P* = 0.95, χ^2^ test). Febrile episodes related to oral cavity infections accounted for 22.6% (37/164) of all febrile patients.

#### Febrile patients associated with odontogenic infections

The types of oral infections associated with febrile episodes are shown in Table 1. Among patients with oral cavity infection-related fevers, 78.4% (29/37) were clinically associated with odontogenic infections, representing 17.7% (29/164) of all febrile patients and 7.1% (29/408) of all patients (Fig. 1A). There was no significant difference in frequency between the AC group (7.7%, 22/285) and the TC group (5.7%, 7/123) (*P* = 0.46, χ^2^ test) (Fig. 1B).

**Table 1:** Type of oral infection associated with febrile patients

Type of oral infection	Number of patients (frequency, %)		
	AC (*n* = 285)	TC (*n* = 123)	Total (*n* = 408)
Odontogenic infections	22 (7.7)	7 (5.7)	29 (7.1)
Pericoronitis	9 (3.2)	2 (1.6)	11 (2.7)
Periodontitis (periodontal infection)	7 (2.5)	4 (3.3)	11 (2.7)
Periapical periodontitis	6 (2.1)	1 (0.8)	7 (1.7)
Oral mucositis	3 (1.1)	1 (0.8)	4 (1.0)
Bite wound		1 (0.8)	1 (0.2)
Gingival infection caused by poor hygiene under the pontic of a dental bridge	1 (0.4)		1 (0.2)
Unknown		2 (1.6)	2 (0.5)
Total	26 (9.1)	11 (8.9)	37 (9.1)

Across both treatment groups, the most common odontogenic infections were pericoronitis (2.7%, 11/408), periodontitis (2.7%, 11/408; infection of the periodontal pocket), and periapical periodontitis (1.7%, 7/408; infection at the tooth apex resulting from internal root infection due to dental diseases, such as caries).

### First chemotherapy cycle during which a febrile episode clinically associated with chronic odontogenic infection occurred

**Fig. 2:**
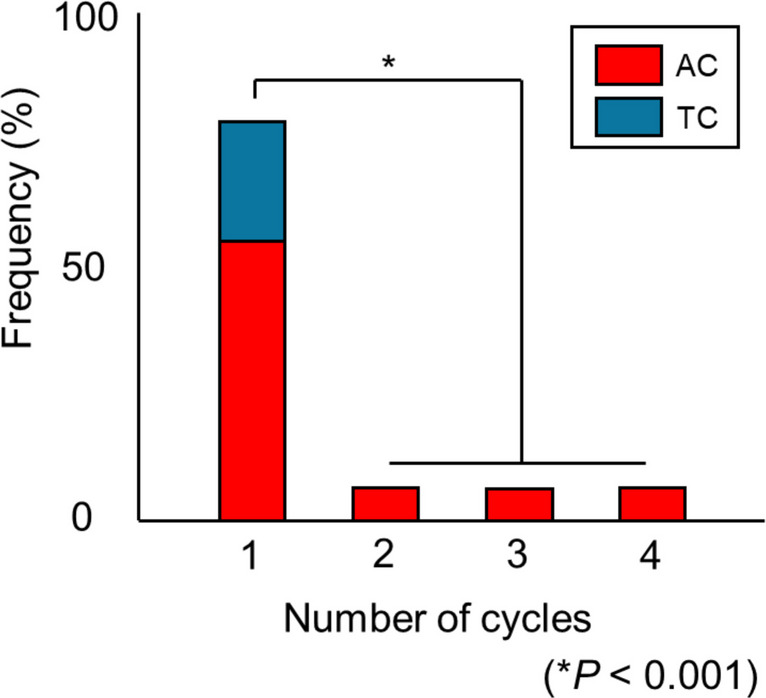
First chemotherapy cycle during which a febrile episode clinically associated with chronic odontogenic infection occurred. The first febrile episode occurred significantly more often in the first cycle (79.3%, 23/29) than in subsequent cycles (second cycle 6.9% [2/29], third cycle 6.9% [2/29], fourth cycle 6.9% [2/29]; total 6.9% [6/87]; *P* < 0.001, Fisher’s exact test, comparing the first cycle with cycles 2–4)

Figure 2 shows the first chemotherapy cycle during which a febrile episode clinically associated with chronic odontogenic infection occurred. The first febrile episode occurred significantly more often in the first cycle (79.3%, 23/29) than in subsequent cycles (second cycle 6.9% [2/29], third cycle 6.9% [2/29], fourth cycle 6.9% [2/29]; total 6.9% [6/87]; *P* < 0.001, Fisher’s exact test, comparing the first cycle with cycles 2–4).

Three patients underwent low-invasive tooth extraction after a febrile episode clinically associated with odontogenic infection, and no further febrile episodes were documented thereafter during chemotherapy. Similarly, in four other patients with febrile episodes clinically judged to be associated with pericoronitis (*n* = 1) or periodontitis (*n* = 3), local minocycline ointment administration together with oral hygiene instruction and management was followed by no further febrile episodes during the subsequent chemotherapy cycles.

### RDI in patients with febrile episodes clinically associated with chronic odontogenic infection

RDI was reduced in 3 patients with febrile episodes clinically associated with chronic odontogenic infection, representing 0.7% of the total cohort (3/408) and 10.3% of patients with febrile episodes clinically associated with chronic odontogenic infection (3/29). RDIs of chemotherapy in the 3 patients were 85%, 90%, and 92.5%, respectively. All reductions in RDI were due to dose reductions and not to treatment delays.

## Discussion

Febrile episodes clinically associated with odontogenic infection occurred in 7.1% of patients receiving AC or TC perioperative chemotherapy for breast cancer. These episodes were significantly more frequent during the first cycle, occasionally resulting in a 7.5%–15% reduction in RDI.

In this study, fever generally developed during the expected neutropenic phase of chemotherapy. In routine clinical practice, FN is often diagnosed clinically even when microbiological confirmation is unavailable. However, in the present retrospective study, FN could not be definitively confirmed in all cases, and attribution of fever to an odontogenic source was based primarily on dental findings and the documented clinical course. Guidelines from the European Organisation for Research and Treatment of Cancer (EORTC) [6], the American Society of Clinical Oncology (ASCO) [7], and the NCCN [4] recommend that prophylactic G-CSFs be used when risk of FN associated with the chemotherapy regimen is ≥ 20% and may be considered when the risk is 10%–20%, particularly when additional patient risk factors are present. The observed frequency of febrile episodes clinically associated with odontogenic infections suggests that such infections may represent a clinically relevant contributor to febrile events during AC or TC chemotherapy.

The overall frequency of febrile episodes was 36.5% in the AC group and 48.8% in the TC group. NCCN classifies AC as intermediate risk for FN (10%–20%) and TC as high risk for FN (> 20%) [4]. A previous study at our institute indicated an FN frequency of 28.3% among patients undergoing TC therapy [11]. As many patients experienced febrile episodes at home, precise neutrophil counts at the time of the fever were often unavailable. It is therefore unclear whether their neutrophil counts at the time of fever met the criteria for FN, which likely contributed to the higher observed incidence compared with previous reports on FN. In the US Oncology Research Trial 9735 (USON9735), a large-scale randomized clinical trial, the incidence of FN in patients less than 65 years old was 2% with AC and 4% with TC [2], reflecting the wide use of prophylactic antibiotics, whereas prophylactic antibiotics and G-CSF were not routinely administered in the present study. Differences in definitions of fever (≥ 38.5 °C oral in USON9735 vs. ≥ 37.5 °C axillary in this study) may also account for the observed discrepancy.

It was notable that the frequency of febrile episodes clinically associated with odontogenic infections did not differ between AC and TC groups, despite higher overall FN risk with TC. The first febrile episode clinically associated with chronic odontogenic infection occurred predominantly during the first cycle, suggesting that even regimens with differing myelosuppressive profiles may trigger fever once a threshold of bone marrow suppression is exceeded.

Maintaining adequate RDI during perioperative chemotherapy is associated with improved DFS and OS, while reductions below 85% are linked to poorer outcomes [12]. In this study, some patients showed a 7.5%–15% reduction in RDI after febrile episodes clinically associated with odontogenic infection. Although final RDIs remained ≥ 85%, early dental intervention and oral management may help minimize treatment compromise.

This study had several limitations. Detailed dental examinations were not performed for all patients prior to chemotherapy, which restricted the precise assessment of preexisting odontogenic infections. In some cases, oral infection was assessed based on referral records and chart review rather than direct examination by the study dentists. Moreover, microbiological confirmation was not systematically available; blood culture data were not consistently obtained at the time of fever, and oral pathogens were not identified in a standardized manner. Therefore, attribution of fever to an odontogenic source was based primarily on dental findings and the documented clinical course rather than definitive microbiological evidence. To provide context, data from the Survey of Dental Diseases conducted by the Japanese Ministry of Health, Labour and Welfare [13] can be used to infer the dental status of a population similar to that included in this study. Among individuals aged 50–54 years, corresponding to the median age of our cohort, the average number of present teeth was 26.9 (out of 28 teeth, excluding third molars). Periodontal assessment revealed that 37.4% had pockets ≥ 4 mm, generally indicative of mild to moderate periodontitis, whereas 3.3% had pockets ≥ 6 mm, suggesting severe periodontitis. Only 2.5% of subjects had decayed teeth, defined as permanent teeth with untreated carious lesions, with an average of 0.7 decayed teeth per individual. These findings suggest a relatively low prevalence of active dental caries in this age group. Data on third molars and apical periodontitis were not available in this national survey. In addition, because patient management was not standardized, the specific effects of dental interventions could not be clearly distinguished from those of other concomitant interventions. Prospective studies are warranted to clarify the causal relationships between odontogenic infections and febrile episodes during chemotherapy.

Preoperative dental treatment is often challenging due to the urgent need to initiate chemotherapy. However, intervention may be feasible during the interval between surgery and postoperative chemotherapy. In this study, the first febrile episode clinically associated with chronic odontogenic infection occurred predominantly during the first chemotherapy cycle. In several patients, no further febrile episodes were documented after dental management, including low-invasive tooth extraction or local periodontal treatment. However, these observations should be interpreted cautiously because of the small number of cases and the retrospective, non-standardized nature of patient management. When patients are suspected to have chronic odontogenic infections, this information should be shared among physicians, nurses, dentists, dental hygienists, pharmacists, and other members of the multidisciplinary healthcare team. Patients should be educated about the importance of oral hygiene during chemotherapy. Although direct evidence linking dental management and patient self-care to a reduction in febrile episodes remains limited, these findings support the potential value of integrating dental assessment and oral care into perioperative management. Prospective studies are warranted to confirm these benefits and establish standardized protocols for timing, scope, and patient guidance.

In conclusion, odontogenic infections should be considered one possible source of febrile episodes during perioperative AC or TC chemotherapy for breast cancer, particularly during the first cycle.

## Data Availability

The raw data supporting the conclusions of this article will be made available by the authors upon reasonable request.
